# The RNA Chaperone Hfq Participates in Persistence to Multiple Antibiotics in the Fish Pathogen *Yersinia ruckeri*

**DOI:** 10.3390/microorganisms9071404

**Published:** 2021-06-29

**Authors:** Iván L. Calderón, María José Barros, Fernanda Montt, Fernando Gil, Juan A. Fuentes, Lillian G. Acuña

**Affiliations:** 1 Laboratorio de RNAs Bacterianos, Departamento de Ciencias Biológicas, Facultad de Ciencias de la Vida, Universidad Andres Bello, Santiago 8370186, Chile; mbarrosgamonal@gmail.com (M.J.B.); fernanda.monttc@gmail.com (F.M.); 2 Microbiota-Host Interactions and Clostridia Research Group, Departamento de Ciencias Biológicas, Facultad de Ciencias de la Vida, Universidad Andres Bello, Santiago 8370186, Chile; fernandogil@unab.cl; 3 ANID-Millennium Science Initiative Program-Millennium Nucleus in the Biology of the Intestinal Microbiota, Santiago 8370186, Chile; 4 Laboratorio de Genética y Patogénesis Bacteriana, Departamento de Ciencias Biológicas, Facultad de Ciencias de la Vida, Universidad Andres Bello, Santiago 8370186, Chile; jfuentes@unab.cl; 5 Interdisciplinary Center for Aquaculture Research (INCAR), Universidad Andres Bello, Viña del Mar 2531015, Chile

**Keywords:** Hfq, sRNA chaperone, persistence, multiple antibiotics, *Yersinia ruckeri*

## Abstract

*Yersinia ruckeri* causes outbreaks of enteric redmouth disease in salmon aquaculture all over the world. The transient antibiotic tolerance exhibited by bacterial persisters is commonly thought to be responsible for outbreaks; however, the molecular factors underlying this behavior have not been explored in *Y. ruckeri*. In this study, we investigated the participation of the RNA chaperone Hfq from *Y. ruckeri* in antibiotic persistence. Cultures of the *hfq*-knockout mutant (Δ*hfq*) exhibited faster replication, increased ATP levels and a more reductive environment than the wild type. The growth curves of bacteria exposed to sublethal concentrations of ampicillin, oxolinic acid, ciprofloxacin and polymyxin B revealed a greater susceptibility for the Δ*hfq* strain. The time-kill curves of bacteria treated with the antibiotics mentioned above and florfenicol, using inoculums from exponential, stationary and biofilm cultures, demonstrated that the Δ*hfq* strain has significant defects in persister cells production. To shed more light on the role of Hfq in antibiotic persistence, we analyzed its dependence on the (p)ppGpp synthetase RelA by determining the persister cells production in the absence of the *relA* gene. The Δ*relA* and Δ*relA*Δ*hfq* strains displayed similar defects in persister cells formation, but higher than Δ*hfq* strain. Similarly, stationary cultures of the Δ*relA* and Δ*relA*Δ*hfq* strains exhibited comparable levels of ATP but higher than that of the Δ*hfq* strain, indicating that *relA* is epistatic over *hfq*. Taken together, our findings provide valuable information on antibiotic persistence in *Y. ruckeri*, shedding light on the participation of Hfq in the persistence phenomenon.

## 1. Introduction

Bacterial persistence is increasingly recognized as one of the major causes of antibiotic treatment failure and recurrence of infections [1,2]. Persisters are a subpopulation of slow-growing or growth-arrested bacterial cells with a decreased susceptibility to bactericidal antibiotics within a susceptible clonal population [3]. Antibiotic persistence is an ability widely described in bacterial species irrespective of whether they possess genes enabling antibiotic resistance [3,4,5]. However, the underlying mechanism of persister production is a complex and still unclear phenomenon that includes multiple systems and pathways such as the (p)ppGpp-mediated stringent response, toxin-antitoxins (TA), SOS response to DNA damage, RpoS-mediated general stress response, modulation of energy metabolism, and drug efflux pumps [1,6,7]. In recent years, some studies have explored the participation of the small RNA (sRNA) chaperone Hfq in antibiotic persistence, showing differences among bacteria and conditions [5,7,8]. Hfq is conserved in a wide range of bacteria and regulates several physiological processes to maintain intracellular homeostasis [9]. The importance of Hfq under stressful environments is evident from the pleiotropic effects observed when the *hfq* gene is inactivated in different bacteria. The *hfq* gene disruption produces a decreased growth rate, cell filamentation, and increased sensitivity to stresses and alters motility and biofilm formation [9]. The regulatory role of Hfq on antibiotic persistence is still a poorly explored field and differs from one species to another [5,7,8]. For instance, *Escherichia coli* Δ*hfq* persister cells formation capacity increases when challenged by ampicillin [8], and defective persistence increases following exposure to gentamicin, levofloxacin or cefotaxime [7]. In the case of the fish pathogen *Aeromonas veronii*, *hfq* deletion plays a negative role in persister cells formation under the treatments of tetracycline, cefotaxime, ciprofloxacin and chloramphenicol [5].

*Yersinia ruckeri* is a facultative, Gram-negative pathogen responsible for the enteric redmouth disease (ERM), a severe septicemic disease that mainly affects salmonids and, consequently, the fish farming industry. In recent years, the number of outbreaks provoked by this pathogen has substantially increased in the aquaculture sector [10], affecting fish in all development stages and exhibiting higher mortality in adult specimens [11]. The ability of *Y. ruckeri* to survive in fish farms is based on its adaptation to disadvantageous environmental conditions such as antibiotic exposure [12]. The most commonly used antibiotics worldwide, such as prophylaxis and to treat ERM outbreaks, include oxytetracycline, florfenicol, oxolinic acid, amoxicillin and sulfadiazine, in combination with trimethoprim [13,14,15,16]. In vitro studies have demonstrated that *Y. ruckeri* can develop resistance to oxolinic acid, oxytetracycline and the potentiated sulphonamide [17]. However, to our knowledge, studies focused on antibiotic persistence in this pathogen have not been reported to date. To investigate the regulatory role of *Y. ruckeri* Hfq in antibiotic persistence, we compared *Y. ruckeri* wild-type and Δ*hfq* regarding their growth and metabolic status, the susceptibility to sublethal concentrations of bactericidal antibiotics, and persister cells production upon exposure to lethal concentrations of such antibiotics. Furthermore, to better understand the participation of Hfq in antibiotic persistence, its dependence on *relA* ((p)ppGpp synthetase encoding gene) was also analyzed. Our results provide evidence for the participation of Hfq from *Y. ruckeri* in antibiotic persistence and contribute to understanding the phenomenon of antibiotic persistence in this salmonid pathogen.

## 2. Materials and Methods

Bacterial strains and culture conditions: *Y. ruckeri* strains were routinely grown at 26 °C in Trypticase soy broth (TSB) or Trypticase soy agar (TSA). The strains and plasmids used in this work are listed in Table 1. pCM433 was donated by Christopher Marx (Addgene plasmid # 15670; http://n2t.net/addgene:15670, accessed on 29 May 2021, RRID:Addgene_15670), and pDiGc was donated by Sophie Helaine and David Holden (Addgene plasmid # 59322; http://n2t.net/addgene:59322, accessed on 29 May 2021; RRID:Addgene_59322).

Construction of gene deletions mutants: The deletion mutant strains Δ*relA* and Δ*hfq* Δ*relA* (double mutant) were constructed as we previously described for Δ*hfq* [18], using an unmarked allelic exchange method based on the sacB-dependent suicide plasmid pCM433 [19]. Briefly, the *relA* gene sequence of *Y. ruckeri* CD2 was amplified by PCR with forward and reverse primers containing the *Nde*I and *Bgl*II restriction sites, respectively. Both the PCR product and the pCM433 plasmid were digested with *Nde*I and *Bgl*II and then ligated. The plasmid carrying *relA* gene was then transformed into *Y. ruckeri* CD2 to obtain the Δ*relA* strain. Transformants were first selected on TSA plates supplemented with chloramphenicol (0.075 mg mL^−1^) and then plated on TSA supplemented with 5% sucrose for the selection of sucrose-resistance clones. To select for recombinants that have excised the vector, we performed a negative selection on TSA plates supplemented with chloramphenicol (0.075 mg mL^−1^). The Δ*relA* strain was confirmed by PCR, using primers flanking the substitution site. To obtain the double mutant strain Δ*relA* Δ*hfq*, the pCM433 plasmid carrying the *relA* gene was transformed into the single Δ*hfq* strain and the selection of the double knock-out mutant was performed as described above. For the complementation assays, *hfq* was cloned with its promoter into pBR322. The PCR product obtained with primers Hfq_pBR322_EcoRI_Fw and Hfq_pBR322_HindIII_Rv was digested with EcoRI and HindIII and then cloned into pBR322, in the respective restriction sites, to yield pHfq. Specific primers used in this work are listed in Supplementary Table S1.

Minimum inhibitory concentration (MIC) and minimum bactericidal concentration (MBC) determination: Bacteria were grown overnight in TSB medium, washed three times with sterile phosphate-buffered saline (PBS), diluted in fresh TSB medium and aliquoted (100 μL) into the wells of sterile microtitre plates (10^8^ CFU mL^−1^ in each well). MIC and MBC were determined using serial 10-fold microdilution of the antibiotics in TSB medium. The lowest concentration of antibiotics that inhibited growth (measured as the OD_600_) by at least 50%, relative to growth in the absence of antibiotics, was defined as the MIC. MBC was defined as 99.9% killing of the starting inoculum.

Persister assay. Persistence was determined by measuring the viable bacteria as colony-forming units (CFU) per mL. The cultures were prepared from different inoculums: exponential phase cells (OD_600_ of 0.3), stationary phase cells (OD_600_ of 0.9) and bacteria recovered from biofilm. For biofilm assay 100 μL of an overnight culture was inoculated in a 24-well polystyrene microtiter plate containing 900 μL of TSB. The plates were incubated without agitation at 26 °C for 24 h and then washed three times with PBS. Adherent bacteria were recovered by sonication in PBS and resuspended in fresh TSB. The OD_600_ was measured to estimate total cell number for each sample: exponential, stationary and biofilm recovered cells. Sample were adjusted to 10^8^ cells/mL in TSB and exposed to antibiotics (1 mg mL^−1^ ampicillin, 0.05 mg mL^−1^ ciprofloxacin, 0.01 mg mL^−1^ florfenicol, 0.05 mg mL^−1^ oxolinic acid or 1 mg mL^−1^ polymyxin B), at desired time points and washed in PBS before plating on TSA without antibiotics to determine CFU count.

Growth curves: Bacteria were cultured in TSB media at 26 °C overnight, diluted into 100 mL of fresh TSB medium at a ratio of 1:100, and incubated with shaking at 200 rpm until stationary phase. When required, the cultures were treated with sublethal concentrations of each antibiotic (ampicillin, oxolinic acid, ciprofloxacin, florfenicol and polymyxin B). The OD_600_ was measured every 30 min until stationary phase of growth (16 h), using a Synergy H1 microplate reader (Biotek). The experiments were repeated three times.

Intracellular ATP levels and NAD^+^/NADH ratios: Bacteria were grown in TSB until OD_600_ 0.9 and the quantification of ATP and NAD^+^/NADH was performed with the ATP Fluorometric Assay Kit and NAD^+^/NADH quantification kit, respectively (BioVision Research Products, Milpitas, CA, USA), as previously described [18]. The ATP levels and NAD^+^/NADH ratios were normalized to the bacterial cell concentration.

Measurement of bacterial replication by fluorescence dilution: The procedure was performed as previously described [18] with some modifications. Bacterial strains carrying pDiGc plasmid [21] were aerobically grown overnight at 26 °C in TSB medium supplemented with 4% arabinose to allow production of red fluorescent protein. These bacteria were purified by cell sorting using FACSAria III (Becton Dickinson, New Jersey, USA). Bacteria (6 × 10^6^ cells) were resuspended in 2 mL of TSB and incubated at 26 °C for 12 h. Aliquots of 200 µL were taken at different times (0, 3, 6, 9 and 12 h), and bacteria were analyzed using a FACSCalibur flow cytometer (Becton Dickinson). Data were analyzed using FlowJo 8.6.3 software.

Statistics: The data was statistically analyzed with Student’s *T*-test. Values of *p* < 0.05 were considered statistically significant.

## 3. Results

### 3.1. Hfq Deletion Results in Bacteria with Faster Growth and More Active Metabolism

**Figure 1:**
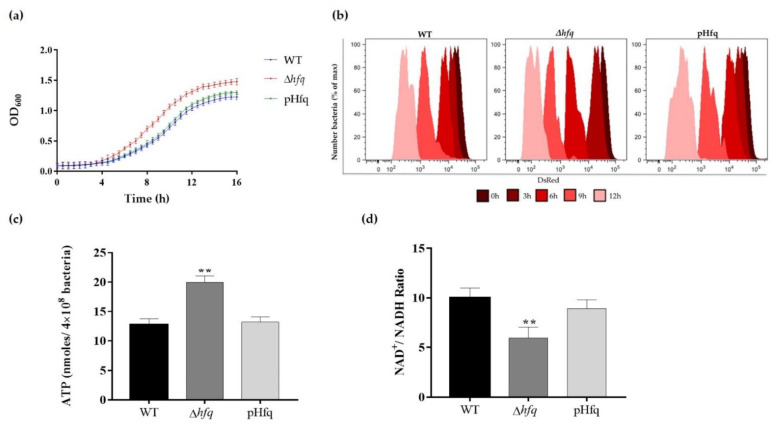
Growth curves, bacterial replication and metabolic status of *Y. ruckeri* strains. (**a**) Wild-type (WT), ∆*hfq* and pHfq (complemented) strains were grown in TSB medium and OD_600_ was measured at different time points. Data represent the means ± standard deviations (*n* = 3). (**b**) Flow cytometric detection of DsRed and EGFP fluorescence at different time points from the WT, ∆*hfq* and pHfq strains (carrying pDiGc plasmid) cultured in TSB medium for 12 h (*n* = 30,000 events analyzed at each time point). WT, ∆*hfq* and pHfq strains were grown in TSB to OD_600_ of 0.9 and both the intracellular ATP levels (**c**) and NAD^+^/NADH ratio were determined (**d**). Asterisks represent statistical differences with respect to the WT strain (** *p* < 0.001). Data represent the means ± standard deviations (*n* = 3).

Since Hfq is a global regulator that impacts growth and metabolism in bacteria [22,23], we first analyzed these processes in *Y. ruckeri* cultures. For this purpose, a mutant strain deficient in this regulatory protein was constructed (∆*hfq* strain) prior to analyzing the growth curves, replication profiles and metabolic status. We observed that *Y. ruckeri* ∆*hfq* exhibited an accelerated bacterial growth in comparison with the wild-type strain (Figure 1a). The bacterial replication in the culture medium was accurately determined by a fluorescence dilution (FD) assay based on a replication reporter system, as previously described [18]. The FD results supported the analysis of growth curves, showing that the Δ*hfq* strain replicates faster than the wild type, displaying a more accelerated profile of red fluorescence dilution as compared to the wild-type strain (Figure 1b). In accordance with the growth and replication phenotypes, the intracellular levels of ATP from stationary cultures of the ∆*hfq* strain were higher than that of the wild-type (Figure 1c). In agreement with a more active metabolic state, *Y. ruckeri* Δ*hfq* also showed a lower NAD^+^/NADH ratio than the wild-type (Figure 1d), evidencing a more reductive intracellular environment. The pHfq complemented strain showed phenotypes comparable to the wild-type strain. All these results suggest that Hfq modulates bacterial proliferation and energy metabolism.

### 3.2. Y. ruckeri Δhfq Exhibits Increased Susceptibility to Sublethal Concentrations of Different Antibiotics

**Figure 2:**
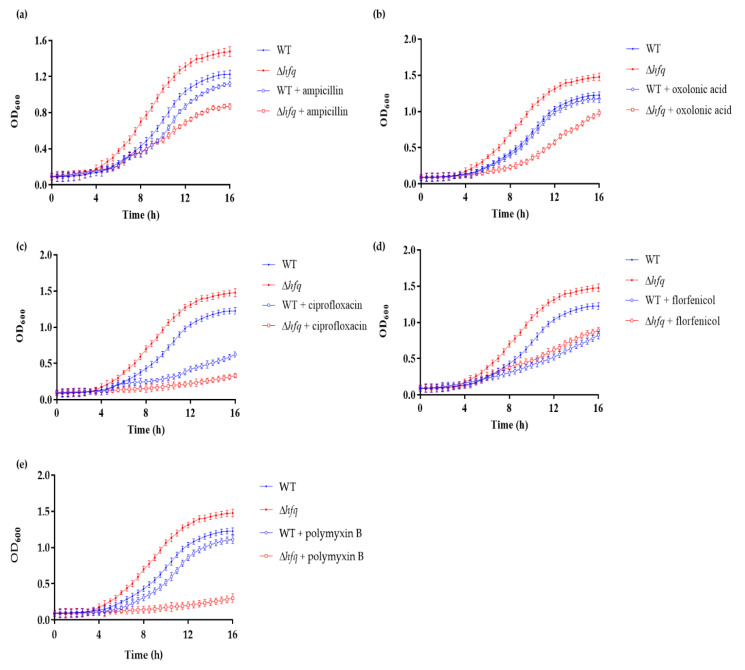
Growth curves of the WT and ∆*hfq* strains exposed to sublethal concentrations of antibiotics. WT and ∆*hfq* strains were grown in TSB medium supplemented with 0.01 mg/mL ampicillin (**a**), 0.0001 mg/mL oxolinic acid (**b**), 0.001 mg/mL ciprofloxacin (**c**), 0.001 mg/mL florfenicol (**d**) and 0.002 mg/mL polymyxin B (**e**). OD_600_ was measured every 30 min until the bacteria reached the stationary phase (16 h). Data represent the means ± standard deviations (*n* = 3).

To gain better insights into the role of Hfq in antibiotic response in *Y. ruckeri*, we first evaluated the susceptibilities of the Δ*hfq* and wild-type strains by determining the minimal inhibitory concentration (MIC) and minimum bactericidal concentration (MBC) with different bactericidal antibiotics. The deletion of *hfq* increased the susceptibility 10-fold compared to all the tested antibiotics, except for florfenicol (Table 2). Then, the strains were grown in the presence of sublethal concentrations of the antibiotics, confirming that the Δ*hfq* strain presented enhanced susceptibility toward most of the antibiotics tested, except for florfenicol (Figure 2a–e). The highest susceptibility was observed for polymyxin B, suggesting that Hfq is critical for regulating outer membrane homeostasis. Overall, these results revealed that the efficiency of the antibiotics on the bacterial growth was higher with the rapidly dividing Δ*hfq* cells having high metabolic rates.

### 3.3. Hfq Is Required for Antibiotics Persistence in Y. ruckeri

**Figure 3:**
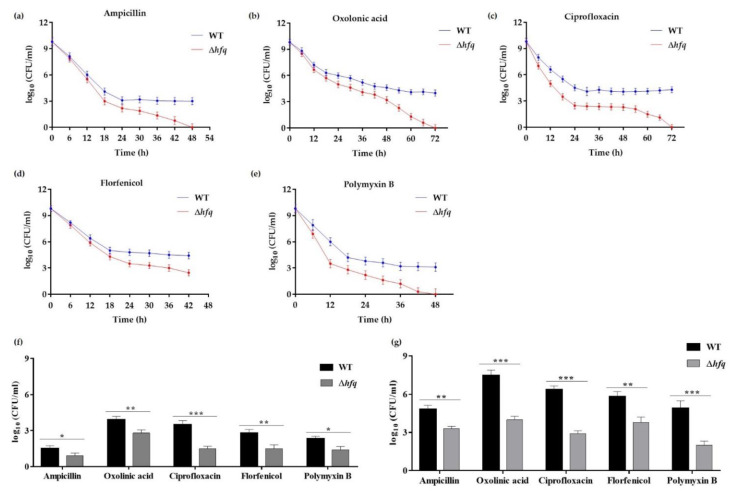
Time-kill curves and persister cells production of the WT and ∆*hfq* strains exposed to antibiotics. Stationary cells of the WT and Δ*hfq* strains were exposed to 1 mg mL^−1^ ampicillin (**a**), 0.05 mg mL^−1^ oxolinic acid (**b**), 0.05 mg mL^−1^ ciprofloxacin (**c**), 0.01 mg mL^−1^ florfenicol (**d**) or 1 mg mL^−1^ polymyxin B (**e**), and the survival of persister cells was measured as CFUs per 1 mL at different time points. Exponential (**f**) and biofilm resuspended (**g**) cells of the WT and Δ*hfq* strains were exposed to 1 mg mL^−1^ ampicillin, 0.05 mg mL^−1^ oxolinic acid, 0.05 mg mL^−1^ ciprofloxacin, 0.01 mg mL^−1^ florfenicol or 1 mg mL^−1^ polymyxin B for 24 h, and the survival of persister cells was measured as CFUs per 1 mL at 24 h. Asterisks represent statistical differences with respect to the wild-type strain (* *p* < 0.01; ** *p* < 0.001; *** *p* = 0.0001). Data represent the means ± standard deviations (*n* = 3).

To analyze the role of Hfq in antibiotic persistence of *Y. ruckeri*, susceptibility of stationary-phase cells of the Δ*hfq* and the wild-type strains was examined during the exposure to lethal concentrations of bactericidal antibiotics (time-kill curves) as an indicator for persister cells production. Survival was significantly diminished in the Δ*hfq* strain upon treatment with ampicillin for 48 h (3-fold), oxolinic acid for 72 h (4-fold), ciprofloxacin for 72 h (~4-fold), polymyxin B for 48 h (~3-fold) and florfenicol for 42 h (~2-fold) (Figure 3a–e), indicating that the persister cells production is affected in the Δ*hfq* strain compared with the wild type. Since the age of inoculum strongly influences persister cells formation [6,24,25], we analyzed whether Hfq also mediates the persister cells production (24 h) from bacteria at exponential phase of growth (OD_600_ of 0.3). We observed that the survival of the Δ*hfq* strain was also decreased, but to a lesser extent, compared to the wild type (Figure 3f). Since persisters have also been described as dormant subpopulations arising within bacterial biofilms that are characteristically highly tolerant to antibiotics [26], we also analyzed the time-kill curves using resuspended cells that had just been sampled from 24 h-biofilms from both Δ*hfq* and the wild-type strain. As previously reported in other persister assays with cells suspension from biofilm cultures [27], we observed that cells harvested from biofilms produce more persister cells than those from liquid cultures. However, persister cells production in the Δ*hfq* strain was also decreased for all the antibiotics treatments for 24 h, in comparison with the wild-type (Figure 3g). These results indicate that Hfq was involved in antibiotic persistence in bacteria harvested from liquid and biofilm cultures.

### 3.4. The Effect of Hfq on Persister Cells Production Is Further Aggravated in the ΔrelA ((p)ppGpp synthetase) Genetic Background

**Figure 4:**
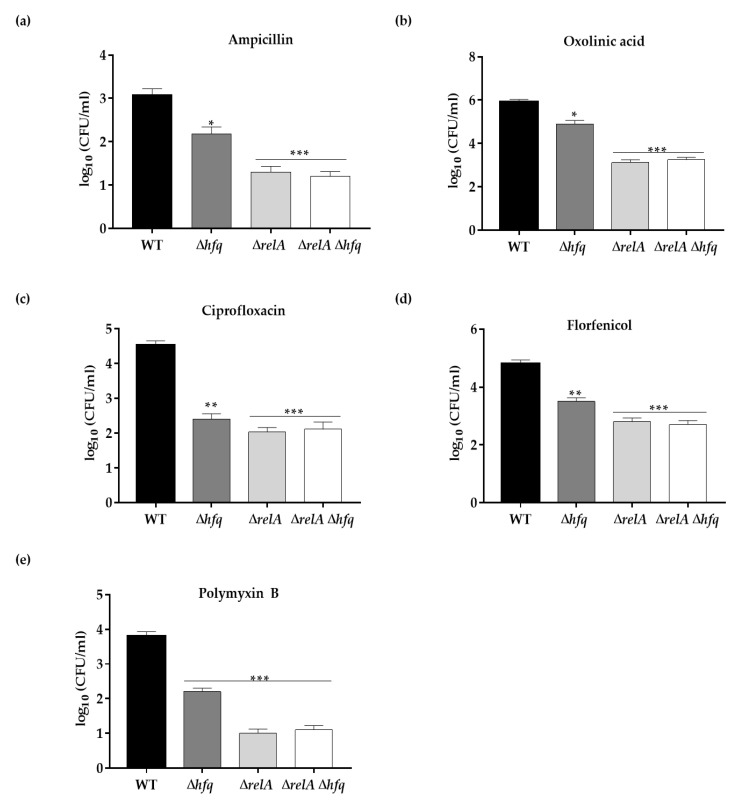
Persister cells production of the Δ*relA* and Δ*relA* Δ*hfq* strains exposed to antibiotics. Stationary cells of the WT, Δ*hfq*, Δ*relA* and Δ*relA* Δ*hfq* strains were exposed to 1 mg mL^−1^ ampicillin (**a**), 0.05 mg mL^−1^ oxolinic acid (**b**), 0.05 mg mL^−1^ ciprofloxacin (**c**), 0.01 mg mL^−1^ florfenicol (**d**) or 1 mg mL^−1^ polymyxin B (**e**) for 24 h, and the survival of persister cells was measured as CFUs per 1 mL at 24 h. Asterisks represent statistical differences with respect to the wild-type strain (* *p* < 0.01; ** *p* < 0.001; *** *p* = 0.0001). Data represent the means ± standard deviations (*n* = 3).

To better understand the role of Hfq in antibiotic persistence and since it is well known that (p)ppGpp is a key regulator of persistence [28], we evaluated whether the role of Hfq in persister cells production is dependent on RelA, the (p)ppGpp synthetase described as a central regulator of persistence [29]. To address this purpose, we constructed the single and double knockout mutants for the *relA* gene, namely, the Δ*relA* and Δ*relA* Δ*hfq* strains. We determined the persister levels of the single and double knockout mutants Δ*relA*, Δ*hfq*, Δ*relA* Δ*hfq* and the wild-type strain from stationary phase cultures treated with lethal concentrations of ampicillin (1 mg mL^−1^), ciprofloxacin (0.05 mg mL^−1^), florfenicol (0.01 mg mL^−1^), oxolinic acid (0.05 mg mL^−1^) and polymyxin B (1 mg mL^−1^) for 24 h. We found that the single-gene knockout of *relA* affected the persister level to the same extent as the double-gene knockout mutant Δ*relA* Δ*hfq* but to a greater extent than the single-gene knockout of *hfq*, for all the antibiotics treatments (Figure 4a–e). These results indicates that the *relA* gene is epistatic over *hfq* regarding this phenotype.

**Figure 5:**
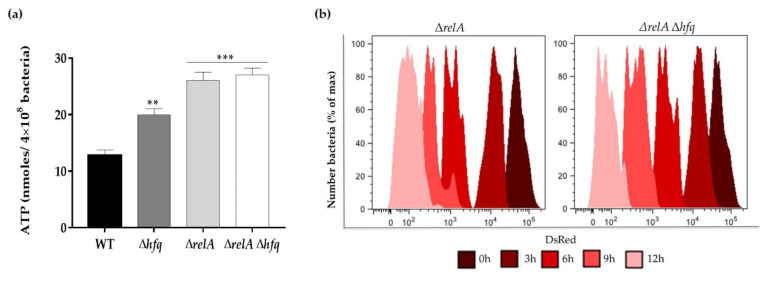
Bacterial replication and metabolic status of the Δ*relA* and Δ*relA* Δ*hfq* strains. (**a**) WT, Δ*hfq*, Δ*relA* and Δ*relA* Δ*hfq* strains were grown in TSB to OD_600_ of 0.9 and the intracellular ATP levels were determined. Asterisks represent statistical differences with respect to the WT strain (** *p* < 0.001; *** *p* = 0.0001). Data represent the means ± standard deviations (*n* = 3). (**b**) Flow cytometric detection of DsRed and EGFP fluorescence at different time points from the Δ*relA* and Δ*relA* Δ*hfq* strains (carrying pDiGc plasmid) cultured in TSB medium for 12 h (*n* = 30,000 events analyzed at each time point).

To further investigate the interaction between the *hfq* and *relA* genes, the replication in bacterial cultures and the ATP levels of stationary phase cells, from the single and double Δ*relA* backgrounds strains, were determined as described above. We observed that the faster replication and hypermetabolic status previously detected in the Δ*hfq* strain was increased in the absence of *relA*, in both the single and double deletion backgrounds (Δ*relA* and Δ*relA* Δ*hfq* strains), confirming the epistatic effect observed above (Figure 5a,b). Together, these data indicate that the regulatory effect of RelA on persistence, energy metabolism and bacterial proliferation exceeds that of Hfq.

## 4. Discussion

Vaccination against the major serotypes of *Y. ruckeri* is used in most fish farms worldwide, although this practice is not effective in all cases [10], and the emergence of outbreaks has substantially increased since 2003 [11]. This situation has awakened interest to investigate the molecular mechanisms of virulence [10,18], and the antimicrobial resistance in *Y. ruckeri* isolates from aquaculture [13,14,30,31]. However, studies focused on persister cells production, i.e., the formation of cells highly tolerant to killing by antibiotics, have not been reported to date. In the present study, we investigated the contribution of the sRNA chaperone Hfq from *Y. ruckeri* in persistence to multiple antibiotics, using an isolate from a salmon farm in Chile.

Among the most frequently used antimicrobials to treat ERM and other bacterial diseases affecting aquaculture, we can mention oxytetracycline and florfenicol [13,14,15,16]. Other antibiotics used, but to a lesser extent, are oxolinic acid and amoxicillin [15,16]. ERM outbreaks have been commonly related to the bacterial resistance to these antibiotics [13,17,32]. However, persister cells could also be responsible for outbreaks and the recalcitrance of infections, since they remain viable and repopulate the fish tanks forming biofilms when the level of antibiotics drops [26]. Persisters are thought to be less sensitive to antimicrobials because they are not undergoing cellular processes that antibiotics can affect, i.e., these cells are in an arrest-growth state that enables antibiotic tolerance. Hfq is a global regulator that impacts growth and metabolism in many bacteria, including some human pathogens of the *Yersinia* genus [22,23,33]. However, contrary to what is commonly observed in those bacteria, the *hfq* deletion in *Y. ruckeri* resulted in faster growth than the wild-type (Figure 1a), as well as in a more active metabolic state (Figure 1b–d). These results suggest global modulatory roles for Hfq in the restraint of growth and/or energy metabolism in this bacterium. To our knowledge, similar phenotypes have been previously described in this and other species lacking Hfq-dependent sRNAs [18,34,35], but not in *hfq* knockout mutants from other species, where the phenotypes are even dissimilar. For example, in *Aeromonas veronii*, the knockout of the *hfq* gene caused a slower growth rate at the exponential phase and produced less cell mass at the stationary phase [5]. By contrast, the knockouts of *hfq* in other species such as *Y. pestis* [33], *E. coli* [36] and *S*. Typhimurium [37] were also affected on growth but in *Y. pseudotuberculosis* displayed no effect [33]. Although Hfq is highly conserved among bacteria, homologs from different species may exhibit variations in their functions intimately linked to the lifestyle or niches that bacteria inhabit.

As we expected, given that reduced ATP levels have been associated with diminished antibiotic efficacy and vice versa [38,39], the *Y. ruckeri* Δ*hfq* strain was more susceptible to most of the antibiotics tested under the sub-lethal concentrations evaluated (Figure 2, Table 2). Differential increased susceptibility to different classes of antibiotics has also been described in other *hfq* deletion strains such as *Pseudomonas aeruginosa* [40], *E. coli* [41], *Salmonella* Typhimurium [42] and *Proteus mirabilis* [43], indicating that the regulatory functions of Hfq enable bacteria to adapt to multiple antibiotic challenges. Similarly, some studies have demonstrated that some Hfq functions involved in antibiotic susceptibility are indirectly regulated via multiple sRNAs [44,45], thus explaining the pleiotropic phenotypes observed in *hfq* knockouts of different species.

For its part, the knowledge on the impact of Hfq on antibiotic persistence is still limited. Previous studies have reported discrepant results regarding the persister cells formation in *hfq* knockouts backgrounds. While in *A. veronnii* the deletion of *hfq* reduced the persister cells formation at different extents, under treatment with ciprofloxacin, cefotaxime, chloramphenicol and tetracycline [5], in *E. coli,* the deletion of *hfq* increased persister cells production by 11-fold under treatment with ampicillin [8]. Another study with the uropathogenic *E. coli* strain UIT89 revealed that the sRNA RyhB mediates the persister cells production to levofloxacin, cefotaxime and gentamicin in an Hfq-independent manner; however, the single deletion of *hfq* also has a detrimental effect on persister cells formation [7]. Together, our results and the data obtained from other studies reporting differential phenotypes for *hfq* mutants highlight the complexity of the persistence phenomenon governed by many different and heterogeneous molecular mechanisms and factors. Among these factors, the bacterial genetic background that evolved from their different lifestyles also played a pivotal role.

Since greater ATP levels are predictive of bactericidal antibiotic efficacy, and taking into account that the downregulation of transcription of genes involved in energy production and the ATP depletion are conditions that explain bacterial tolerance to antibiotics [38,39,46], we hypothesized that the *Y. ruckeri* Δ*hfq* strain would be affected in persister cells formation. Our persister assays were conducted with five classes of antibiotics with bactericidal properties, whose choice was also based on their aquaculture usage. As we expected, the *hfq* deletion mutant showed defects in persister cells formation to different extents for all the antibiotics tested. This result is consistent with previous reports where Δ*ryhB* (sRNA) and Δ*phoU* (regulator for phosphate metabolism) mutants from *E. coli* with hyperactive metabolic states have significant defects in persistence [7,24]. A previous study showed that metabolic stimulation can be used to eliminate persisters [47], suggesting that Hfq might be a potential target for anti-persisters strategies.

As the central stringent-response regulator, RelA represents the main (p)ppGpp synthetase, whose deficiency affects the production of persister cells [48]. We observed a significant reduction in persister cells production when the Δ*relA* mutant was treated with the five antibiotics (Figure 4). The reduction in persister levels of the Δ*relA* strain was comparable to that of the Δ*relA* Δ*hfq* strain for each antibiotic treatment, being more drastic than the effect observed in the Δ*hfq* strain, indicating that *relA* is epistatic over *hfq*. This epistatic effect was further confirmed when the ATP levels were measured from stationary cultures without antibiotic treatments, as well as the replication analysis (Figure 5). As mentioned above, RelA is the major synthetase of (p)ppGpp, the second messenger that plays a crucial role in persister formation. By controlling diverse physiological processes, (p)ppGpp leads to dormancy or slow growth in bacteria [29,49]. In addition, it is known that, in *E. coli*, RelA fulfills functions independent of its (p)ppGpp synthetase activity. By interacting with Hfq, RelA stimulates the regulatory activity of the Hfq-dependent sRNA RyhB to arrest growth during iron depletion [50,51]. Based on this information, we hypothesize that RelA has a regulatory effect on Hfq by direct protein–protein interaction, and it also plays a role with the (p)ppGpp synthetase activity, which could explain the above-mentioned epistatic effect. However, the possibility of a mechanism involving RelA, Hfq and sRNAs in antibiotic persistence in *Y. ruckeri* needs to be further studied.

In summary, our results provide evidence that the sRNA chaperone Hfq from *Y. ruckeri* restrains the growth and energy metabolism and mediates persistence to multiple antibiotics. In addition, the RelA-mediated persistence displayed by several bacterial pathogens was also evidenced in *Y. ruckeri*. Thus, this study contributes to understanding the phenomenon of antibiotic persistence in this salmonid pathogen and will aid in the rational design of novel strategies to prevent or treat ERM outbreaks.

## Figures and Tables

**Table 1:** Bacterial strains and plasmids used in this study.

Strain	Characteristics	Reference
*Y. ruckeri* CD2	Wild-type strain of *Y. ruckeri*	[18]
Δ*hfq*	*Y. ruckeri* CD2 lacking *hfq* gene	[18]
Δ*relA*	*Y. ruckeri* CD2 lacking *relA* gene	This study
Δ*relA* Δ*hfq*	*Y. ruckeri* CD2 lacking *realA* and *hfq* genes	This study
**Plasmid**	**Genotype**	**Reference**
pCM433	Ap^R^, Cm^R^, Tc^R^, oriT, tet, cat, *sacB* and ColEI ori; broad-host-range sacB-based allelic exchange vector	[19]
pCM433-*hfq*	*hfq* region of *Y. ruckeri* CD2 cloned into pCM433	This study
pCM433-*relA*	*relA* region of *Y. ruckeri* CD2 cloned into pCM433	This study
pBR322	ApR, TcR, ColEl Ori.	[20]
pHfq	*hfq* region of *Y. ruckeri* CD2 cloned into pBR322	This study
pDiGc	*bla* GFP pBAD DsRed ori f1 Amp^R^	[21]

**Table 2:** MIC and MBC measurements for wild-type (WT), Δ*hfq* and pHfq (complemented) strains in TSB medium supplemented with antibiotics.

Antibiotic	MIC (mg mL^−1^)			MBC (mg mL^−1^)		
	WT	Δ*hfq*	pHfq	WT	Δ*hfq*	pHfq
Ampicillin	1	0.1	1	1	0.1	1
Oxolinic acid	0.005	0.0005	0.005	0.05	0.5	0.05
Ciprofloxacin	0.003	0.003	0.003	0.003	0.3	0.003
Florfenicol	0.01	0.01	0.01	0.01	0.1	0.01
Polymyxin B	2	0.2	2	2	0.2	2

## Supplementary Materials

The following are available online at https://www.mdpi.com/article/10.3390/microorganisms9071404/s1, Table S1: Primers (Oligos) used in this study.
